# *Australia and New Zealand Health Policy*: a new journal

**DOI:** 10.1186/1743-8462-1-1

**Published:** 2004-11-17

**Authors:** Stephen J Duckett

**Affiliations:** 1 Professor of Health Policy La Trobe University, Victoria, 3086, Australia

## Abstract

*Australia and New Zealand Health Policy *is a new journal which aims to promote debate and understanding about contemporary health policy developments in Australia and New Zealand. Although there are other international journals focussing on health policy, there are no Australian or New Zealand journals with this focus.

One of the aims of *Australia and New Zealand Health Policy *is to focus on contemporary critiques and contemporary developments. Accordingly an e-journal format is particularly appropriate. *Australian and New Zealand Health Policy *is an open access journal which means that all articles will be freely and universally accessible online which, amongst other things, means that all articles will be freely and universally accessible online without any barriers to access, which increases their visibility.

## Editorial

Welcome to *Australia and New Zealand Health Policy *a new journal which aims to promote debate and understanding about contemporary health policy developments in Australia and New Zealand. Health policy is regularly in the media and is a high profile issue at election times. In Australia the health system has been characterised by conflicts over values and policy choices over the decades. So pervasive is this conflict that Sax entitled his 1984 book about health services, "A Strife of Interests" [1]. Health policy in New Zealand has also had a turbulent time over the past decade [2,3]. Health policy changes in Australia and New Zealand are thus ripe for analysis. *Australia and New Zealand Health Policy *aims to provide a prestigious venue for analysis and critique of health policy in the two countries.

## Why a new journal?

Although there are other international journals focussing on health policy, there are no Australian or New Zealand journals with this focus. Other related-area local journals are medical, public health or hospital-related. Although the local journals publish occasional policy articles, this area is not their principal interest, nor are they necessarily the journals which policy-oriented academics or policy practitioners scan to keep abreast of developments.

The absence of a health policy journal serving Australia and New Zealand has long-term consequences for the development of systematic analysis of and research into health policy. One consequence is that there is no forum where health policy developments are documented and tracked, a lacuna which precludes cumulative analyses of trends. *Australia and New Zealand Health Policy *will address this by publishing annual reviews of policy developments.

As one of the aims of *Australia and New Zealand Health Policy *is to focus on contemporary critiques and contemporary developments, an e-journal format is particularly appropriate. Debate will also be stimulated by providing for 'Comment' on published articles, in a way analogous to a letters column.

*Australia and New Zealand Health Policy *is a peer reviewed journal. In keeping with its policy-applied focus, articles will be refereed by two referees, preferably one with a strong practitioner background, such as currently or recently employed in a policy role in a health authority, and one from an academic background. At least one of the referees will have substantive content knowledge relating to the article. Articles published in *Australia and New Zealand Health Policy *will be listed in PubMed and permanently archived in PubMed Central as well as certain other national archives.

## Journal scope

*Australia and New Zealand Health Policy *contributes to understanding of health policy development and practice with a particular focus on Australia and New Zealand. It welcomes submissions which:

• Review and critique contemporary health policy issues;

• Identify major trends in health policy and emerging policy issues, including new evidence about the effect of policy changes;

• Identify impacts of health services research on new policies;

• Identify major public policy and governance trends and their application to health policy;

• Analyse contemporary health policy themes which cut across a range of policy areas;

• Report on international policy developments and new international comparisons of health policy involving Australia and/or New Zealand; or

• Critique contemporary health policy developments.

## Open Access

*Australia and New Zealand Health Policy *is an Open Access journal, which means:

• All articles will be freely and universally accessible online without any barriers to access, which increases their visibility.

• You and your peers will be free to print out copies of your article, email it to colleagues, and post it on the web because of the BioMed Central copyright and license agreement.

Open access journals are funded by article processing charges rather than journal subscriptions. The costs are therefore borne by the authors, their institutions of from their grants. That is, all access to journals is free to readers via the web (BMC online-only journals). Authors from institutional supporters are exempt from authorship charges. The institutional supporters pay a sliding scale based on the number of staff and postgraduate students in biomedical sciences.

Institutional subscription has a number of benefits. In addition to the direct benefits in terms of waived author fees, there are public policy benefits in supporting an open access journal regime such as Biomed Central. Open access journals are one mechanism for putting pressure on regular journal publishers to moderate their price increases.

Unfortunately there are no New Zealand institutional subscribers to BioMed Central at present, which means that New Zealand authors will face article processing charges, although no article processing charges will be payable on manuscripts submitted in the first six months following the launch of the journal. Article processing charges are also usually regarded as a legitimate charge against research grants. In the medium term, alternative arrangements, such as institutional support, should be encouraged, although after this time the editor-in-chief will be able to grant a limited number of discretionary processing charge waivers.

## The first articles

*Australia and New Zealand Health Policy *commences its publication program with a series of articles which describe and evaluate health policy developments in Australia in 2003. Responses or commentaries on these articles would be welcome. It is the aim of the Editorial Board to encourage a cluster of articles at the start of each year about health policy developments in Australia and New Zealand in the previous calendar year. Authors who have an interest in reviewing contemporary developments are encouraged to submit manuscripts on these themes early in the New Year so that the articles can contribute in a timely way to health policy debate in the two countries.

S.J.Duckett

Editor-in-Chief
